# Genetic diversity and divergence among native and translocated populations of the golden flathead goby *Glossogobius aureus* (Gobiiformes: Gobiidae) in Philippine lakes

**DOI:** 10.1371/journal.pone.0293076

**Published:** 2023-12-14

**Authors:** Onaya P. Abdulmalik-Labe, Andrew Jason L. Eduardo, Jonas P. Quilang

**Affiliations:** 1 Institute of Biology, College of Science, University of the Philippines Diliman, Quezon City, Philippines; 2 Biology Department, College of Natural Sciences and Mathematics, Mindanao State University, Marawi City, Philippines; CIFRI: Central Inland Fisheries Research Institute, INDIA

## Abstract

The golden flathead goby *Glossogobius aureus* is a native species in the Philippines, Australia, Japan, Taiwan, and many other countries in Asia. In the Philippines, it is an important food fish as it is commonly caught in major lakes. In this study, a total of 307 specimens morphologically identified as *G*. *aureus* were sampled from nine major lakes in the Philippines and were sequenced for their mitochondrial *cytochrome b* (*cyt b*) gene. Two hundred sixty of the 307 *cyt b* sequences had sequence similarities of ≥ 99% with *G*. *aureus* reference sequence in GenBank, while the remaining 47 (all from Lake Lanao) had sequence similarities of only 95% and were thus designated as *Glossogobius* cf. *aureus* and treated as a separate population. The sequences were then analyzed to examine the pattern of genetic diversity, relatedness, divergence, and demographic history among native and translocated populations of the species. Twenty-nine haplotypes were recovered, of which four haplotypes were shared among three to seven populations. Only one haplotype each was found in the native population in Lake Buhi and translocated population in Lake Paoay. Low haplotype and low nucleotide diversities were found for the populations in Laguna de Bay, Lanao, Bato, Buhi, Paoay, and Sebu lakes, which indicate founder event for the introduced populations in Lanao, Paoay, and Sebu lakes and recent genetic bottleneck for the native populations in Laguna de Bay, Bato, and Buhi. In contrast, high haplotype but low nucleotide diversities were found for the native populations of Taal, Naujan, and Buluan lakes, signifying a recent bottleneck followed by population expansion. Pairwise *F*_ST_ values showed generally large (*F*_ST_ = 0.168–0.249) to very large (*F*_ST_ = 0.302–1.000) genetic divergence between populations except between Laguna de Bay and Lake Bato, Laguna de Bay and Lake Buhi, and Lake Bato and Lake Buhi populations, which showed nonsignificant genetic differentiation. Lake Buluan and Lake Sebu populations showed moderate genetic differentiation (*F*_ST_ = 0.098). Neutrality tests showed significant negative Tajima’s *D* and Fu’s *F*_*S*_ values only for the population from Laguna de Bay, which suggests that the population is undergoing expansion. These results are important for establishing scientifically sound strategies for effective conservation and sustainable exploitation of *G*. *aureus* in the Philippines.

## Introduction

The golden flathead goby *Glossogobius aureus* Akihito and Meguro 1975 (Gobiiformes: Gobiidae) is an amphidromous fish that is native to the Philippines, Taiwan, Japan, Thailand, Singapore, Australia, Malaysia, Cambodia, Papua New Guinea, Laos, and Indonesia [1–6]. *Glossogobius aureus* belongs to the most speciose genus in the suborder Gobioidei (gobies) [5], a large suborder in the order Gobiiformes [7]. In the Philippines, the most diverse group among the freshwater fishes are the gobies [8, 9], in which *G*. *aureus* is the most widely distributed and the most abundant goby species in many lakes [10]. *Glossogobius aureus* is a commercially important species. Unlike many species of gobies in which the adults are quite small, *G*. *aureus* can grow to a large size (up to 30 cm), and it has been found to have a high growth rate [11], thus it has the potential to be developed for aquaculture. The adult gobies, mainly *G*. *aureus*, are considered a delicacy in the Philippines. They are an important source of livelihood among residents surrounding many major lakes in the Philippines, such as in Laguna de Bay, Lake Lanao, Lake Taal, and Lake Naujan. Gobies are sold either as fresh or dried fish. In addition, the goby fry are a component of the *ipon* fishery [12, 13], a collective term for the fry of several goby species in the Philippines. *Ipon* fishery was once reported to be an important source of livelihood among the fisherfolk of the northern coast of Luzon [12]. Goby fry are used as food and as the main ingredient in making fish pastes [14]. *Glossogobius* sp. was one of the predominant fish species in Laguna de Bay in the 1950s [15] and in Lake Naujan in the 1970s and 1980s [16]. Over the years, a general decline in the volume of fish catch of *Glossogobius* sp. has been observed by Mercene and Nasino [15] in Laguna de Bay and by Ismail et al. [17] in Lake Lanao. Freshwater gobies (mainly *G*. *aureus*) ranked as the 7th most abundant inland species caught by volume in the Philippines [18]. However, this volume decreased to 2,916.74 metric tons in 2021, so its rank slid to 11^th^ [19].

Although *G*. *aureus* is widely distributed in East and Southeast Asia and serves as an important food fish [11], the current knowledge about this species is limited to its feeding habits [20], scale shape [21], population biology [22], and reproductive traits [11]. To date, there are no studies yet on the genetic diversity and population genetic structure of *G*. *aureus* in the Philippines. As an important food fish and a candidate species for aquaculture, conservation and management of this fish is of prime importance. There is a need to study the population genetics of this species to guide policymakers and concerned government agencies in the proper management and conservation of this important biological resource. High genetic diversity in a population would mean greater resilience to fluctuating environmental conditions [23] and greater ability for long-term persistence [24]. Thus, maintenance of high levels of genetic variation in natural populations is desirable for conservation [25]. On the other hand, low genetic variation would render the population more vulnerable to extinction due to inbreeding depression and failure to adapt to environmental changes [24]. Several genetic studies have investigated the levels of genetic diversity and divergence among native and introduced populations of fishes [26–30]. The general expectation is native populations tend to have higher genetic diversity than introduced populations [30]. This is because of the founder effect in introduced populations. Introduced populations carry only a portion of the genetic composition of the source population [26]. These founder populations are exposed to other factors that reduce their number, thus further reducing their genetic diversity. High genetic diversity is expected to be observed in native populations of *G*. *aureus* in Laguna de Bay, Taal, Naujan, Buluan, Bato, and Buhi lakes. Additionally, low to high genetic diversity is expected among the translocated populations of *G*. *aureus* in Lake Lanao, Paoay, and Sebu, depending on the number of individuals introduced and how well the introduced populations were able to adapt to their new environments. Knowledge of the population genetic structure of *G*. *aureus* in the Philippines can serve as a basis for determining the number of independent management units (IMUs). The identification of distinct IMUs will enable fisheries managers to develop targeted conservation and management strategies based on the unique characteristics of each population.

Several mitochondrial DNA (mtDNA) markers, including *cytochrome b* (*cyt b*) gene, have been used in many studies to investigate the genetic diversity within and between populations of fishes and other animals. These mtDNA markers are often chosen for population genetic studies because they are easier to use and relatively cheaper compared to other molecular markers. In addition, mtDNA lacks repair mechanisms, resulting in an increased rate of evolution and making it useful in identifying major evolutionary lineages [31]. The mtDNA *cyt b* gene, in particular, has been used in several fish population genetic studies. Sousa-Santos et al. [32] used mtDNA *cyt b* to determine the factors that affect the pattern and levels of genetic diversity in the different populations of native Portuguese cyprinids. Mitochondrial DNA *cyt b* was also used by Ju et al. [33] to investigate whether Kuroshio Current can act as a barrier or facilitator in shaping the population structure of barcheek goby *Rhinogobius giurinus* in East Asia. Faulks et al. [34] also used the mtDNA *cyt b* to assess the genetic diversity and structure of the critically endangered endemic Edgbaston goby *Chlamydogobius squamigenus* from Great Artesian Basin springs at Edgbaston, Queensland, Australia. Their study aimed to assist management agencies in establishing relocated populations of *C*. *squamigenus*. Khosravi et al. [35] used mtDNA *cyt b* to molecularly identify *Carassius* species in Iran and investigate the origin and pathways of introduction of the non-native *Carassius* species. Genetic variation analysis was performed by Jose et al. [36] on the threatened Malabar pufferfish *Carinotetraodon travancoricus* using mtDNA *cyt b*. Abbas et al. [37] studied the demographic structure of five populations of yellowcheek *Elopichthys bambusa* in Yangtze River using mtDNA *cyt b*. Recently, Gong et al. [38] used mtDNA *cyt b* to investigate the levels of genetic diversity and genetic structure in populations of the endemic cyprinid *Schizopygopsis younghusbandi* in the Yarlung Tsangpo River drainage, Tibetan Plateau. They also examined the correlation between the genetic structure of *S*. *younghusbandi* and landscape determinants such as longitude and elevation.

This study aimed to determine the genetic diversity, relatedness, and divergence among native and translocated populations of *G*. *aureus* from nine major lakes in the Philippines. The genetic data from this study can be used in formulating possible conservation measures in the future and for selecting possible sources of broodstock for aquaculture.

## Materials and methods

### Description of lakes included in this study

Laguna de Bay (14.38 N, 121.25 E) is located in Calabarzon region in Luzon Island, the largest island of the Philippines. It is bounded by Laguna, Rizal, and Metro Manila provinces. It is the largest lake in the Philippines. It has a surface area of 911–949 km^2^ and a maximum and average depth of 7.3 m and 2.8 m, respectively. It has an elevation of less than 2 m above sea level. The lake was originally part of Manila Bay before a blockage was formed by the movement of the West Marikina Valley Fault [39]. Despite this blockage, the lake is still connected to Manila Bay via the Pasig River. Specimens used in this study were collected from Binangonan and Tanay, Rizal.

Lake Lanao (7.88 N, 124.25 E) is located in the province of Lanao del Sur in the northern part of Mindanao Island. It is the 2^nd^ largest lake in the Philippines. It has a total surface area of 357 km^2^, a maximum depth of 112 m, and an elevation of 702 m above sea level [40]. The lake is fed by four large rivers and is drained by the Agus River, the lake’s only connection to the sea, which is now impassable to migrating fishes due to the power plants installed in the river. Specimens used in this study were collected from Marawi City, Marantao, Taraka and Ditsaan-Ramain, Lanao del Sur.

Lake Taal (14.01N, 120.97E), formerly known as Bombon Lake, is also located in Calabarzon in Luzon Island, specifically in Batangas, a province bordering the southwest of Laguna province. It is the 3^rd^ largest lake in the Philippines. It has a surface area of 236.9 km^2^, an elevation of 2.5 m above sea level, and a mean and maximum depth of 90 m and 189 m, respectively [41]. It is a caldera lake that formed as a result of numerous destructive volcanic eruptions as well as other geological processes [42]. Historically, the lake was described by Medina in 1630 to be salty and very deep [43, 44], but 200 years later, Hill reported the lake to be brackish [43]. The violent eruption of Volcano Island in 1754 formed a dome between Lake Taal and Balayan Bay [45]. This dome diverted the Pansipit River to its current position [45]. The original river channel’s depth, length, and width were then reduced [45, 46]. Since Lake Taal was no longer broadly connected to Balayan Bay, there was a gradual decrease in the salinity of the lake’s water, and eventually, it became a freshwater lake. Samples of *G*. *aureus* in the lake were collected from San Nicolas and Talisay, Batangas.

Lake Naujan (13.17N, 121.33E) is situated in the northeastern part of Mindoro Island in the province of Oriental Mindoro. It is geographically shared by Naujan, Victoria, Socorro, and Pola municipalities. Mindoro is an isolated island surrounded by seawater. Lake Naujan is about 81.25 km^2^ wide, making it the 5^th^ largest lake in the Philippines [47]. Lake Naujan has a depth that ranges from 3 m to 45 m [46] and has an elevation of 20 m above sea level [48]. It was created when a chain of volcanoes formed and blocked an embayment on the northeastern corner of Oriental Mindoro [49]. The lake has one outlet, the Butas River, which serves as passage for the migratory species that move between Lake Naujan and Tablas Strait. Samples used in this study were collected from Bayani, Naujan, Oriental Mindoro.

Lake Buluan (6.64N, 124.82E) is found in the central part of Mindanao Island and bounded by the provinces of Sultan Kudarat and Maguindanao. It is about 130 km away from Lake Lanao. It is 61.34 km^2^ wide and considered the 6^th^ largest lake in the Philippines [47]. It is a shallow lake with a maximum depth of 5.5 m [50]. It is a relic of an ancient sea arm that formerly covered the entire central basin of Mindanao at least 70,000 years ago [50]. The lake basin was created by damming the Marbel River with lahar from the Parker and Matutum volcanoes [49]. Samples used in this study were collected from the Sultan Kudarat’s side of the lake.

Lake Bato (13.33N, 123.37E) and Lake Buhi (13.45N, 123.52E) are both located in the Bicol peninsula in the southern part of Luzon. Lake Bato has an area of 28 km^2^, while Lake Buhi has an area of 17 km^2^; they rank as the 8^th^ and 10^th^ largest lakes in the Philippines, respectively [47]. Both lakes are shallow at about 8 m in depth [47]. The distance between the two lakes is about 24 km. The two lakes are interlinked; they are connected to another lake, Lake Baao-Bula, through different tributaries of the Bicol River [51]. During peak flooding, Lake Bato and Lake Baao-Bula combine to form a continuous body of water [51] but not with Lake Buhi due to its higher elevation. Lake Buhi is a young lake that formed in 1641 when a strong earthquake caused the side of Mt. Iriga to collapse and form a natural dam on Barit River [44, 49]. Samples from Lake Bato were collected from the municipality of Bato’s side of the lake while samples from Lake Buhi were collected from Poblacion Buhi, Camarines Sur.

Lake Paoay (18.12N, 120.54E) is situated in the province of Ilocos Norte in the northwestern part of Luzon. It has a surface area that ranges from 4 km^2^ to 4.4 km^2^ and a depth that ranges from 5 m to 12.5 m [52]. Lake Paoay has neither a tributary nor an outlet [52]. It was formed when sand dunes blocked the mouth of an embayment along Ilocos Norte’s coastline [49]. Samples used in this study were collected from two sites: Suba and Nanguyudan, Paoay, Ilocos Norte.

Lake Sebu (6.23N, 124.71E) is located in the Municipality of Lake Sebu, South Cotabato, in the south-central region of Mindanao. It has a surface area of 3.54 km^2^ and an average and maximum depth of 5 m and 57 m, respectively [53]. Lake Sebu was formed when a prehistoric large-magnitude earthquake caused the deposition of blocky sediments on the valley of a southern tributary of Lanon River [49]. The samples were collected from Poblacion, Lake Sebu, South Cotabato.

### Collection and processing of samples

**Fig 1:**
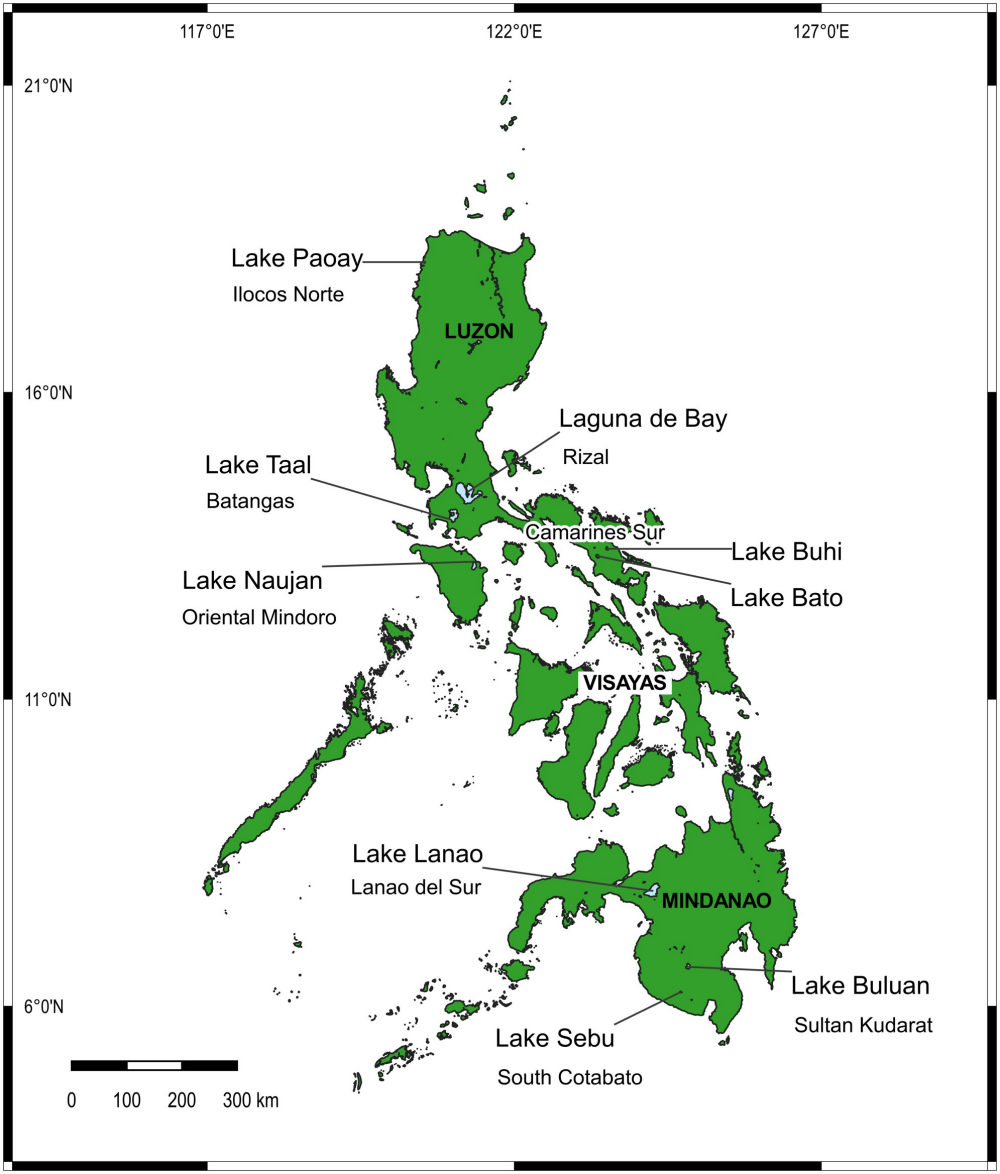
Map of the Philippines showing the location of the sampling sites. The map was drawn using Geographic Information System (QGIS version 3.14π) Open Source Geospatial Foundation Project downloaded from http://qgis.osgeo.org. The base map of the Philippines, including administrative areas and inland water shapefiles, was obtained in vector format from free and open source DIVA-GIS site (http://www.diva-gis.org/gdata).

A total of 307 individuals morphologically identified as *G*. *aureus* were obtained from nine lakes (Fig 1) in the Philippines, namely: Laguna de Bay (*n* = 30), Lake Lanao (*n* = 62), Lake Taal (*n* = 30), Lake Naujan (*n* = 30), Lake Buluan (*n* = 30), Lake Bato (*n* = 30), Lake Buhi (*n* = 30), Lake Paoay (*n* = 35), and Lake Sebu (*n* = 30). The map of the Philippines showing the sampling sites (Fig 1) was drawn using Geographic Information System (QGIS version 3.14π) Open Source Geospatial Foundation Project downloaded from http://qgis.osgeo.org [54]. The base map of the Philippines, including administrative areas and inland water shapefiles, was obtained in vector format from free and open source DIVA-GIS site (http://www.diva-gis.org/gdata) [55].

All fish samples were purchased from fish vendors and fishermen in each lake. This study did not require ethical approval because the used fish samples are commonly sold as food fish in the markets and were already dead when purchased. The specimens were identified morphologically based on Akihito and Meguro [1], Hammer et al. [56], and Hoese and Hammer [57]. Furthermore, the molecular delimitation of *Glossogobius* species in each of the lakes was carried out through DNA barcoding and was published in a separate paper (see reference 10 for details). A small piece of muscle tissue behind the pectoral fins was obtained from the right side of each specimen, and the tissues were stored in separate tubes with absolute ethanol at -20°C. The fish were fixed and stored as voucher specimens in 10% formalin and deposited at the Institute of Biology, University of the Philippines, Diliman, Quezon City, Philippines.

### DNA extraction and PCR amplification of the mtDNA *cyt b* gene

Approximately 20 mg of tissue from each specimen was used for DNA extraction using Promega Wizard^®^ Genomic DNA Purification Kit (Madison, WI, USA), InstaGene^™^ Matrix (BIO-RAD) or Vivantis GF-1 Tissue DNA Extraction Kit (Vivantis, Malaysia) following the manufacturers’ protocols. The extracts were stored at -20°C until further use. DNA yield was quantified using NanoDrop^™^ 2000/2000c spectrophotometer.

The mtDNA *cyt b* gene was amplified using the following primers that were designed with Primer3Plus [58]: forward primer LGCYTB2 (5’–AACCACCGTTGTTATTCAACTACA–3’) and reverse primer GCYTB2H (5’–AGGAAGTATCACTCTGGYTTRATATG– 3’). Polymerase Chain Reaction (PCR) was done in 25 μL reaction mixtures with 0.5 μL of 0.05 mM dNTP, 1.25 μL of each primer diluted to 0.01 mM, 2.5 μL of 10x PCR buffer, 0.125 μL of Taq polymerase (5u/ μL), 17.375 μ of ultrapure water and 2.0 μ of DNA template (5.0–25.0 ng/μL). The reactions were run using the following conditions: initial denaturation step of 95°C for 8 min, 35 cycles of 94°C for 30 s, 55°C for 35 s, and 72°C for 2 min, a final extension step of 72°C for 10 min, and 4°C for storage.

PCR products were analyzed by electrophoresis using 1% agarose gel with ethidium bromide. Approximately 800 bp-sized bands were excised from the gel and purified using QIAquick Gel Extraction Kit (QIAGEN, Valencia, CA) or Thermo Scientific^™^ GeneJET Gel Extraction Kit (Fermentas, Burlington, Ontario, Canada). Purified PCR products were then sent to Macrogen, Inc. (Seoul, Korea) for bidirectional DNA sequencing using Applied Biosystems^™^ 3730xl DNA analyzer.

### Data analysis

The consensus sequence of each specimen was assembled, edited, and trimmed using the pregap4 and gap4 tools in the Staden Package [59]. Each consensus sequence was subjected to online National Center for Biotechnology Information (NCBI) BLASTn and BLASTx analyses [60] to confirm if it was indeed from *Glossogobius* species or from contamination. The consensus sequences were submitted to GenBank and were assigned accession numbers MN306344–MN306498, MZ355635–MZ355664, OP086076–OP086097, OQ032698–OQ032789, and OQ551102–OQ551109. All the consensus sequences were aligned using ClustalW multiple alignment as implemented in BioEdit Sequence Alignment Editor version 7.2.6 [61].

Genetic diversity parameters such as haplotype diversity (*h*), nucleotide diversity (*π*), number of variable sites (*S*), and average number of nucleotide differences (*k*) were computed using DNA Sequence Polymorphism version 6.12.03 (DnaSP) [62]. Population structure was evaluated using Fixation index (*F*_ST_) and analysis of molecular variance (AMOVA) in Arlequin 3.5.1.5 [63]. To test for evidence of recent population expansion, Tajima’s *D* [64] and Fu’s *F*_*S*_ [65] neutrality test and pairwise mismatch distribution [66, 67] were calculated using Arlequin 3.51.5 [63]. To visualize population divergence, structure, and connectivity, the median-joining network (MJN) approach [68] was used as implemented in PopART [69]. Phylogenetic inference analysis was conducted using Maximum Likelihood (ML) and was visualized using the MEGA X program [70]. The Akaike Information Criterion (AIC) and Bayesian Information Criterion (BIC) approaches in the program jModeltest 2.1.1 [71] were used to calculate the most suitable model of nucleotide substitution.

## Results

**Fig 2:**
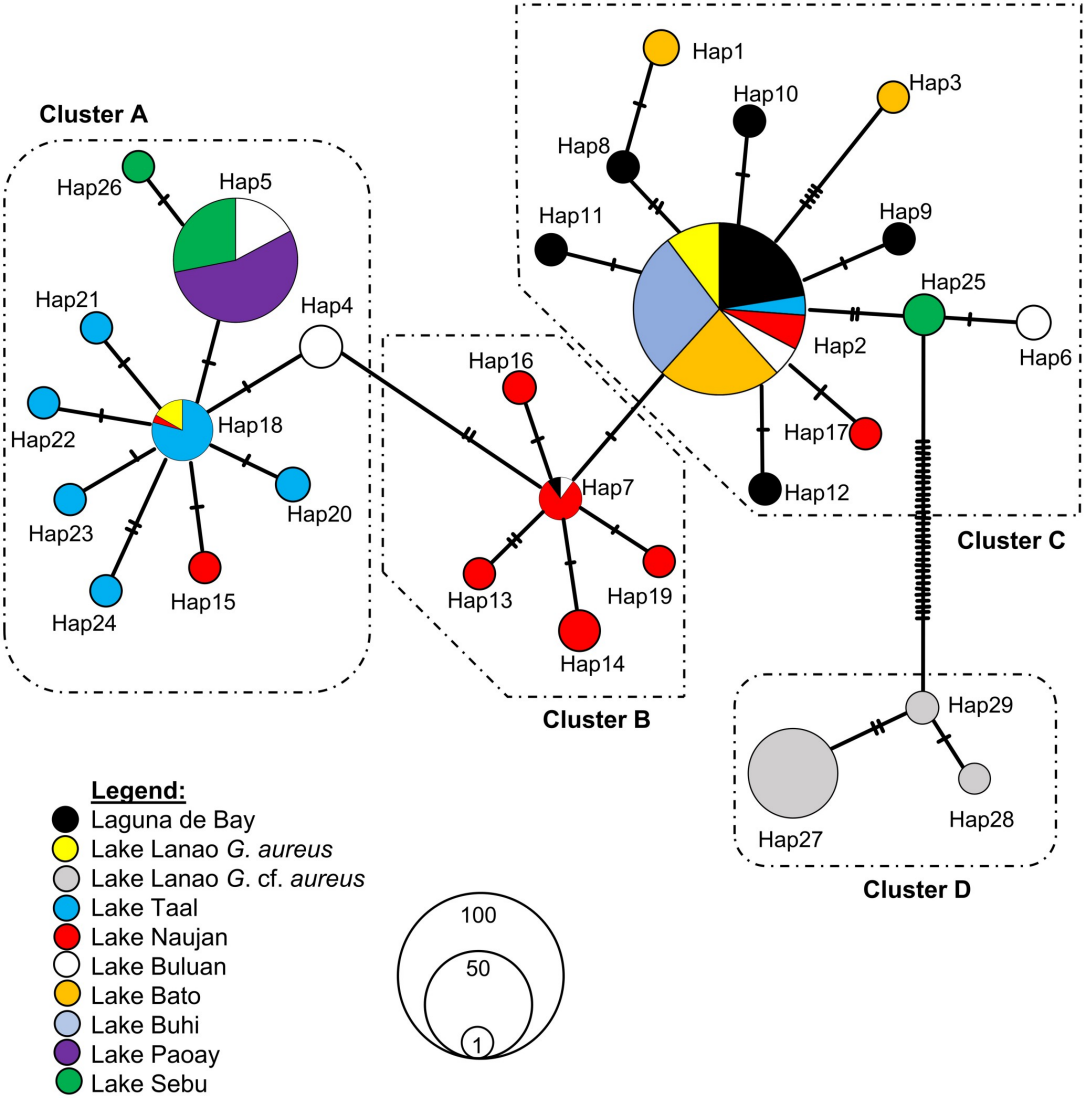
Median-joining haplotype network of *Glossogobius aureus* populations from nine lakes in the Philippines as inferred from mitochondrial *cytochrome b* sequences. Hatch marks on the branches represent the number of mutations between haplotypes. Each circle represents a haplotype, and circle sizes are proportional to the haplotype frequencies.

Nucleotide BLAST (BLASTn) analysis revealed that 260 of the 307 specimens morphologically identified as *Glossogobius aureus* had at least 99% sequence identity with *G*. *aureus* mtDNA *cyt b* in GenBank. These included all the specimens from Laguna de Bay (*n* = 30), Lake Taal (*n* = 30), Lake Naujan (*n* = 30), Lake Buluan (*n* = 30), Lake Bato (*n* = 30), Lake Buhi (*n* = 30), Lake Paoay (*n* = 35), Lake Sebu (*n* = 30) and 15 out of the 62 specimens from Lake Lanao. The remaining 47 specimens from Lake Lanao had the closest similarity to *G*. *aureus* mtDNA *cyt b* in GenBank but with percentage identities of only 95%. Hence, the 47 specimens of this group were designated as *Glossogobius* cf. *aureus* because they fit the morphological description of *G*. *aureus*, but they show genetic divergence from the other specimens based on mtDNA *cyt b* sequence. Nucleotide composition analysis of the 757-bp region of the mtDNA *cyt b* gene showed the following average frequencies: 31.89% for C, 27.68% for T, 24.15% for A, and 16.27% for G; showing obvious anti-G bias. A+T content (51.84%) was higher than C+G content (48.16%). Moreover, a total of 65 polymorphic sites (17 of which are singleton variable sites and 48 are parsimonious informative sites) defined the 29 haplotypes that were detected. Of the 29 haplotypes, a total of 25 (86.21%) were private (i.e., found in a particular population only and not in the other populations). Only four (13.79%) haplotypes were shared among three to seven populations (Table 1, Fig 2). Haplotype 2 (Hap2), the most frequent, was shared among all populations except Lake Paoay and Lake Sebu populations. Haplotype 7 (Hap7) was shared among populations from Laguna de Bay, Naujan, and Buluan lakes. Haplotype 18 (Hap18) was shared among populations from Lanao, Taal, and Naujan lakes. Only one haplotype (Hap5) was recovered from the 35 specimens collected from Lake Paoay; this haplotype was shared with Lake Buluan and Lake Sebu populations. Also, only one haplotype (Hap2) was recovered from Lake Buhi, which was shared with all other *G*. *aureus* populations except Lake Paoay and Lake Sebu. High haplotype diversities, ranging from 0.586 to 0.821, were found only in populations from Taal, Naujan, and Buluan lakes. In contrast, low haplotype diversities, ranging from 0.00 to 0.421, were observed in Laguna de Bay, Lanao, Bato, Buhi, Paoay, and Sebu populations (Table 1). Nucleotide diversities were found to be low in all populations; the values ranged from 0.00% to 0.380% (Table 1).

**Table 1:** Measures of genetic diversity in *Glossogobius aureus* populations from nine lakes in the Philippines as inferred from mitochondrial *cytochrome b* gene sequences.

Population	*n*	No. of haplotypes	No. of unique haplotypes	No. of variable sites (*S*)	Average no. of nucleotide differences (*k*)	Haplotype diversity (*h*)	Nucleotide diversity (π, %)
Laguna de Bay	30	7	5	6	0.40	0.366	0.053
Lake Lanao *G*. *aureus*	15	2	0	4	1.68	0.419	0.221
Lake Lanao *G*. cf. *aureus*	47	3	3	3	0.29	0.124	0.038
Lake Taal	30	7	5	10	1.476	0.586	0.195
Lake Naujan	30	9	6	11	1.66	0.821	0.219
Lake Buluan	30	5	2	8	2.87	0.749	0.380
Lake Bato	30	3	2	7	0.98	0.297	0.130
Lake Buhi	30	1	0	0	0	0.000	0.000
Lake Paoay	35	1	0	0	0	0.000	0.000
Lake Sebu	30	3	2	8	2.66	0.421	0.351
Overall	307	29	25	65	11.69	0.802	1.544

Abbreviation: *n* = number of specimens

AMOVA results showed significant (*P*-value = 0.000) genetic structure among the ten populations of *G*. *aureus*. The results showed that variation among populations (91.44%) was higher than variation within populations (8.56%). These values are supported by both chi-square test (*X*^*2*^: 1240.239, *df* = 252, *P*-value = 0.000) and overall *F*_ST_ value (0.914). *F*_ST_ values were significant for all population pairs except between populations from Laguna de Bay and Lake Bato (*F*_ST_ = 0.045, *P*-value = 0.109), Laguna de Bay and Lake Buhi (*F*_ST_ = 0.000, *P*-value = 0.999), and Lake Bato and Lake Buhi (*F*_ST_ = 0.078, *P*-value = 0.113) (Table 2). Genetic differentiation based on pairwise *F*_ST_ values was found to be significantly moderate (between populations from Buluan and Sebu lakes), large (between populations from Laguna de Bay and Lanao *G*. *aureus*, Lanao *G*. *aureus* and Naujan, Lanao *G*. *aureus* and Buluan, Lanao *G*. *aureus* and Bato, Taal and Buluan, Taal and Sebu, and Paoay and Sebu), and very large (all the remaining population pairs; Table 2).

**Table 2:** Pairwise *F*_ST_ values (below diagonal) and *P*-values (above diagonal) for *Glossogobius aureus* populations from nine lakes in the Philippines as inferred from mitochondrial *cytochrome b* sequences.

**Population**	**Laguna de Bay**	**Lake Lanao *G*. *aureus***	**Lake Lanao *G*. cf. *aureus***	**Lake Taal**	**Lake Naujan**	**Lake Buluan**	**Lake Bato**	**Lake Buhi**	**Lake Paoay**	**Lake Sebu**
Laguna de Bay	-	**0.009**	**0.000**	**0.000**	**0.000**	**0.000**	0.109	0.999	**0.000**	**0.000**
Lake Lanao *G*. *aureus*	0.222	-	**0.000**	**0.000**	**0.003**	**0.005**	**0.002**	**0.013**	**0.000**	**0.000**
Lake Lanao *G*. cf. *aureus*	0.991	0.983	-	**0.000**	**0.000**	**0.000**	**0.000**	**0.000**	**0.000**	**0.000**
Lake Taal	0.758	0.472	0.981	-	**0.000**	**0.001**	**0.000**	**0.000**	**0.000**	**0.000**
Lake Naujan	0.351	0.168	0.977	0.559	-	**0.000**	**0.000**	**0.000**	**0.000**	**0.000**
Lake Buluan	0.492	0.183	0.965	0.176	0.302	-	**0.000**	**0.000**	**0.000**	**0.017**
Lake Bato	0.045	0.189	0.984	0.712	0.328	0.464	-	0.113	**0.000**	**0.000**
Lake Buhi	0.000	0.320	0.995	0.802	0.422	0.531	0.078	-	**0.000**	**0.000**
Lake Paoay	0.964	0.865	0.996	0.611	0.831	0.455	0.918	1.000	-	**0.001**
Lake Sebu	0.659	0.403	0.968	0.249	0.502	0.098	0.626	0.693	0.220	-

Significant values (*P* < 0.05) are shown in bold fonts.

**Fig 3:**
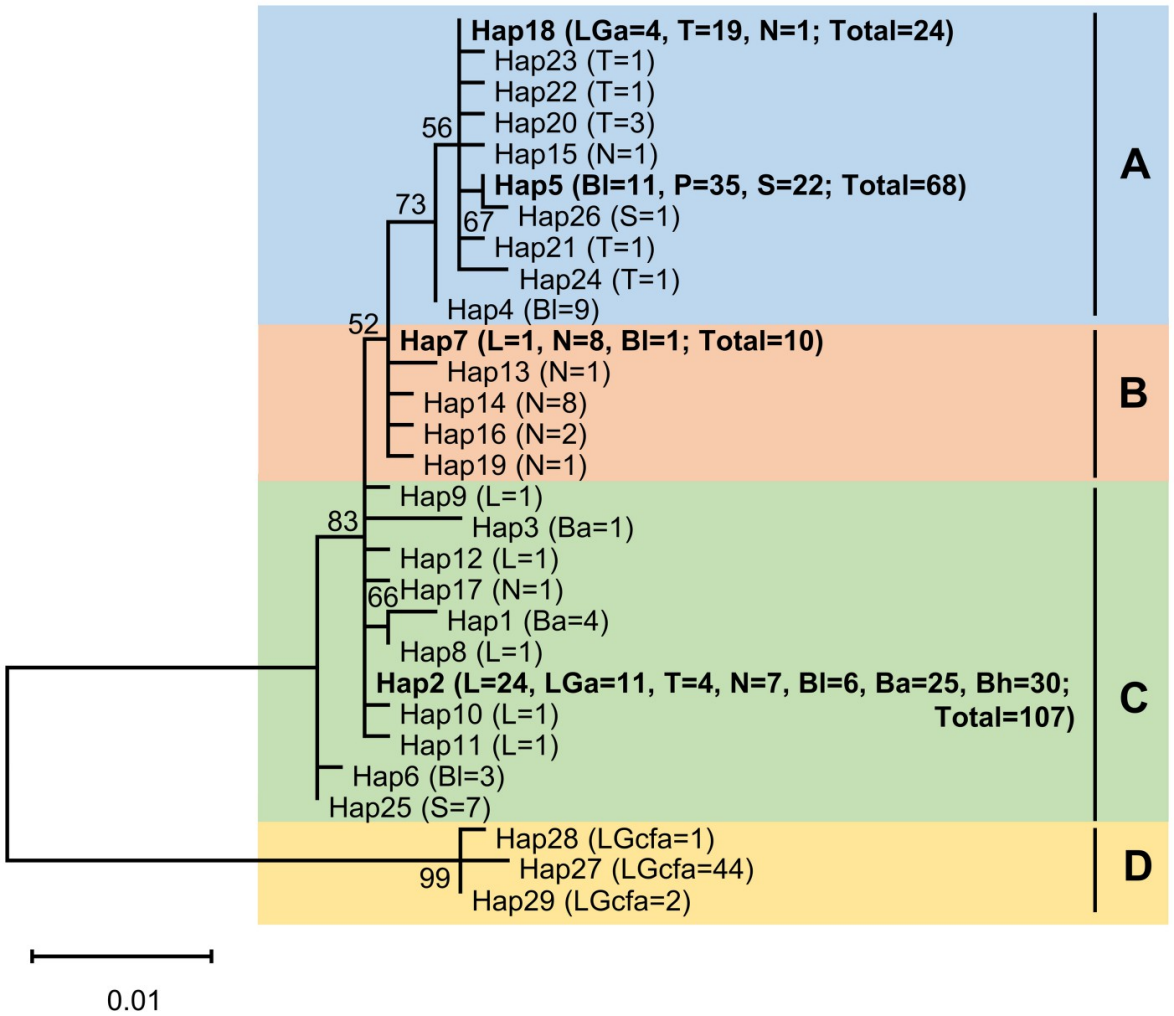
Unrooted maximum-likelihood tree of 29 haplotypes of *Glossogobius aureus* mitochondrial *cytochrome b* sequences from nine lakes in the Philippines. Values on the nodes represent bootstrap support (1000 replicates). Bootstrap support values less than 50% are not shown. The scale bar represents one (1) substitutional change per 100 nucleotide positions. Numbers in parentheses correspond to the number of specimens in each haplotype. Haplotypes (Hap) in bold fonts are shared haplotypes. L = Laguna de Bay, LGa = Lake Lanao *Glossogobius aureus*, LGcfa = Lake Lanao *Glossogobius* cf. *aureus*, T = Lake Taal, N = Lake Naujan, Bl = Lake Buluan, Ba = Lake Bato, Bh = Lake Buhi, P = Lake Paoay, and S = Lake Sebu.

The median-joining network analysis (Fig 2) separated the 29 haplotypes into four clusters, three of which are star-shaped. The first cluster (Cluster A) consists of *G*. *aureus* specimens from Lanao, Taal, Naujan, Buluan, Paoay, and Sebu lakes. The second cluster (Cluster B) consists of *G*. *aureus* specimens from Laguna de Bay, Naujan and Buluan lakes; the third cluster (Cluster C) consists of specimens from all *G*. *aureus* populations except Lake Paoay, while the fourth cluster (Cluster D) is composed of all *G*. cf. *aureus* from Lake Lanao. All the specimens from Lake Paoay were grouped together in Cluster A, while all the specimens from Lake Bato and Lake Buhi were grouped together in Cluster C. This grouping is consistent with the results from maximum likelihood tree constructed using Tamura-Nei (TrN) with gamma distribution model of nucleotide substitution [72] (Fig 3).

**Fig 4:**
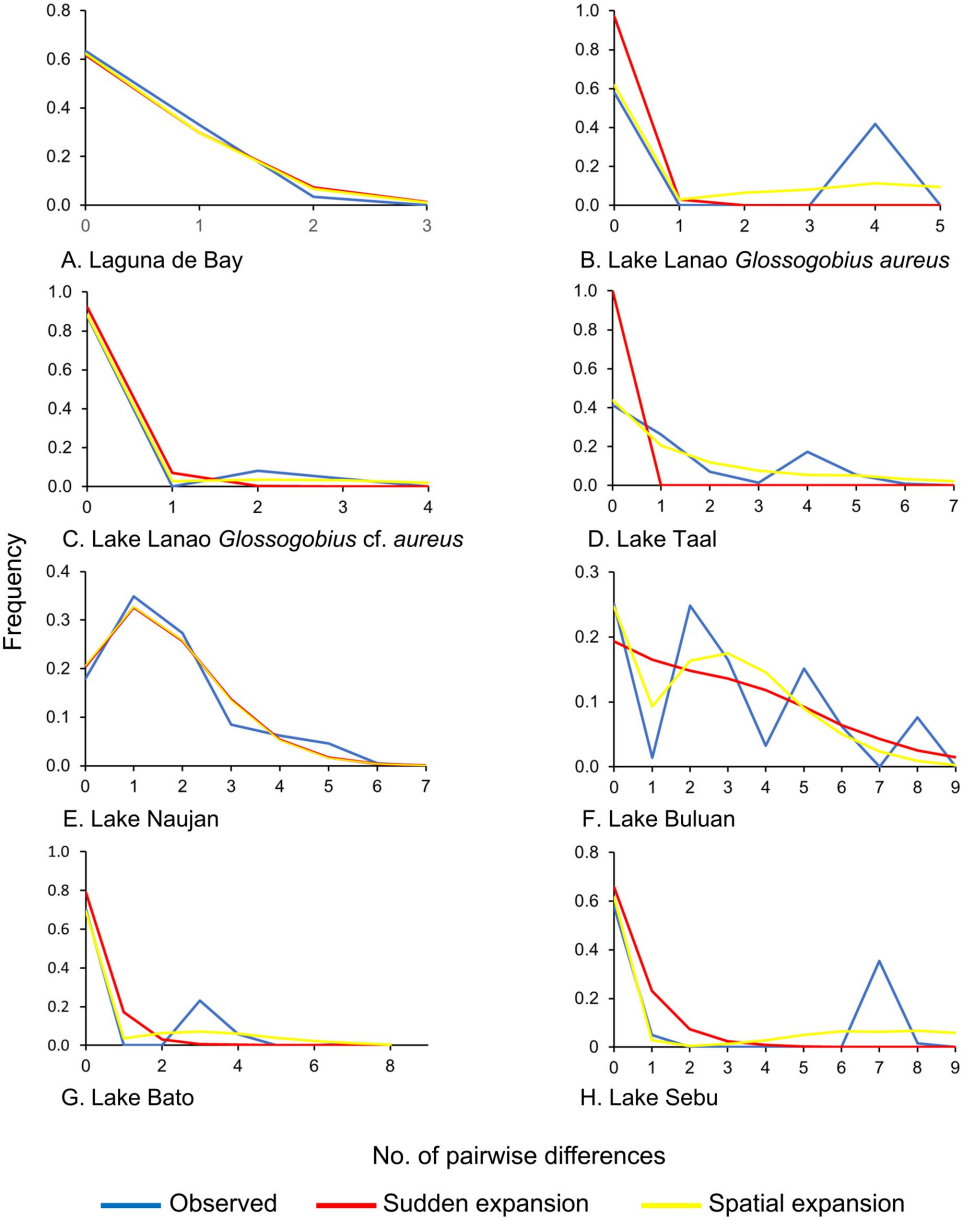
Mismatch analysis plots for eight lacustrine populations of *Glossogobius aureus* in the Philippines based on the mitochondrial *cytochrome b* sequences. There was only one haplotype each for Lake Buhi and Lake Paoay populations, so mismatch analysis could not be done for these two populations.

The results of Tajima’s *D* and Fu’s *F*_*S*_ tests, including associated *P*-values, are shown in Table 3. Significant negative values were observed only in Laguna de Bay population for both Tajima’s *D* and Fu’s *F*_*S*_ tests. This suggests that the mutations in the mtDNA *cyt b* region are not neutral and that the population may be undergoing selection pressure in this gene region. The hypothesis of neutral evolution was rejected for Laguna de Bay population. On the other hand, populations from Lanao, Taal, Naujan, Buluan, Bato, Buhi, Paoay, and Sebu lakes had neutral mutations. The mismatch distribution plots of all populations except Buhi and Paoay populations are shown in Fig 4. Mismatch analysis could not be done for Lake Buhi and Lake Paoay populations because there was only one haplotype in each population. Laguna de Bay and Lake Naujan populations exhibit unimodal plots that closely matched the expected distributions under the sudden and spatial expansion models. Multimodal plots were generated for the populations from Lanao, Taal, Bato, Buluan, and Sebu lakes. Sum of squared deviations (SSD) [73] and the raggedness indices (*r*) [74] under both demographic and spatial expansion models were also calculated for each population, except Lake Buhi and Lake Paoay, and are shown in Table 4. All SSD and raggedness indices were nonsignificant in all populations except the SSD values for sudden expansion model in *G*. *aureus* populations from Lanao, Taal and Bato lakes.

**Table 3:** Neutrality tests for *Glossogobius aureus* populations from nine lakes in the Philippines as inferred from mitochondrial *cytochrome b* sequences.

Population	Tajima’s *D*		Fu’s *F*_*S*_	
	*D*	*P*-value	*F*_*S*_	*P*-value
Laguna de Bay	-2.100	**0.003**	-6.604	**0.000**
Lake Lanao *G*. *aureus*	1.162	0.889	4.144	0.959
Lake Lanao *G*. cf. *aureus*	-1.211	0.080	-0.742	0.210
Lake Taal	-1.315	0.103	-1.412	0.191
Lake Naujan	-1.297	0.087	-2.946	0.053
Lake Buluan	1.285	0.905	2.634	0.899
Lake Bato	-1.309	0.086	1.576	0.829
Lake Buhi	0.000	1.000	NA	NA
Lake Paoay	0.000	1.000	NA	NA
Lake Sebu	0.960	0.860	5.282	0.968

Significant values (*P* < 0.05) are shown in bold fonts.

**Table 4:** Mismatch distribution analysis for eight lacustrine populations of *Glossogobius aureus* in the Philippines as inferred from mitochondrial *cytochrome b* sequences.

Population	Sudden expansion model				Spatial expansion model			
	SSD	*P*-value	*r*	*P*-value	SSD	*P*-value	*r*	*P*-value
Laguna de Bay	0.003	**0.539**	0.181	**0.465**	0.003	**0.292**	0.181	**0.521**
Lake Lanao *G*. *aureus*	0.351	0.00	0.689	**0.927**	0.120	**0.083**	0.689	**0.438**
Lake Lanao *G*. cf. *aureus*	0.014	**0.098**	0.774	**0.758**	0.003	**0.274**	0.774	**0.707**
Lake Taal	0.451	0.000	0.105	**1.000**	0.023	**0.444**	0.105	**0.768**
Lake Naujan	0.005	**0.421**	0.073	**0.353**	0.005	**0.306**	0.073	**0.340**
Lake Buluan	0.052	**0.184**	0.173	**0.085**	0.035	**0.419**	0.173	**0.397**
Lake Bato	0.089	0.039	0.581	**0.499**	0.033	**0.454**	0.581	**0.670**
Lake Sebu	0.169	**0.061**	0.522	**0.380**	0.095	**0.173**	0.522	**0.589**

Nonsignificant values (*P* > 0.05) are shown in bold fonts.

Abbreviations: SSD = Sum of squared deviations, *r* = raggedness indices.

## Discussion

### Status of *Glossogobius aureus* in each lake

In 1975, Akihito and Meguro described a new species of *Glossogobius*, which they named as *G*. *aureus* [1]. In their description of the new species, Akihito and Meguro included *Glossogobius* specimens from various lakes and other collection sites in the Philippines. They were the first ones to report the presence of *G*. *aureus* in Laguna de Bay, Taal, and Lanao lakes. Prior to 1975, only the other large-sized goby *Glossogobius giuris* was reported by Herre to be present in Laguna de Bay [12, 75], Taal [12, 46, 75], Naujan [12, 46, 75], Buluan [12, 75], Bato [12, 75], and Buhi [12, 75] lakes. *Glossogobius giuris* and *G*. *aureus* are morphologically similar and are commonly mistaken for each other [76]. DNA barcoding of *Glossogobius* spp. in Laguna de Bay [10, 77], Lake Taal [10, 76], Lake Naujan [10], Lake Buluan [10], Lake Bato [10] and Lake Buhi [10] revealed that the large-sized *Glossogobius* species in these lakes are *G*. *aureus*. Based on the DNA barcoding studies [10, 76, 77] and previous reports by Herre [12, 75], *G*. *aureus* is considered native to Laguna de Bay, Lake Taal, Lake Naujan, Lake Buluan, Lake Bato, and Lake Buhi. On the other hand, *G*. *aureus* is known to be an introduced species in Lake Lanao and Lake Paoay, as its presence was only detected in the 1960s in Lake Lanao [78] and in 1977 in Lake Paoay [52]. DNA barcoding of *Glossogobius* species in Lake Lanao revealed the presence of two lineages, *G*. *aureus* and *G*. cf. *aureus*, that are separated by a mean pairwise genetic distance of 3.7% based on mtDNA *cytochrome c oxidase I* (*COI*) gene [10]. All the 32 specimens from Lake Lanao (consisting of 10 *G*. *aureus* and 22 *G*. cf. *aureus*) that were DNA barcoded [10] were also used in the present study. The *cyt b* sequences of these 32 specimens generated in this study also formed two lineages that are concordant with the results obtained using *COI* sequences. The *G*. *aureus* population in Lake Sebu is also most likely introduced. Herre conducted extensive surveys of fishes in Mindanao lakes such as Lake Mainit, Lake Lanao, Lake Dapao, Lake Nunungan, and Lake Buluan in the 1920s [12, 79, 80]. He reported the presence of *G*. *giuris* in Lake Buluan [12], a lake about 56.19 km away from Lake Sebu, but did not mention the presence of any *Glossogobius* species in Lake Sebu. The only fish Herre mentioned as present in Lake Sebu is *Chana striata* [75, 80]. Only two publications reported the presence of *Glossogobius* sp., *G*. *celebius* [81] and *G*. *circumspectus* [82], in Lake Sebu. The presence of two unique haplotypes (Hap25 and Hap26) belonging to different haplogroups (Cluster C and Cluster A, respectively) indicates multiple introductions of *G*. *aureus* in Lake Sebu. In this study, we examined the pattern of genetic diversity, relatedness, and divergence among six native (Laguna de Bay, Lake Taal, Lake Naujan, Lake Buluan, Lake Bato, and Lake Buhi) and three translocated lacustrine populations (Lake Lanao, Lake Paoay, and Lake Sebu) of *G*. *aureus* in the Philippines using the mtDNA *cyt b*.

### Patterns of genetic diversity in native and translocated populations

Values obtained for nucleotide and haplotype diversities can be used to infer the demographic history [83] of native and translocated populations of *G*. *aureus* in nine Philippine lakes. High haplotype but low nucleotide diversities were observed in the native populations of *G*. *aureus* from Taal, Naujan, and Buluan lakes. High haplotype diversity and low nucleotide diversity can be attributed to a recent population expansion after a low effective population size caused by bottlenecks or founder events [83]. Large effective population size [84], environmental and habitat heterogeneity [85, 86], broader distributional range, and inter-basin connectivity [28, 32] have all been proposed as explanations for the maintenance of high diversity within populations. *Glossogobius aureus* is an amphidromous fish that spends its planktonic larval stage in marine environment and ascends to freshwater habitat to mature and reproduce. The fact that the species inhabit ecosystems with high environmental heterogeneities may likely account for the high levels of haplotypic diversity observed. Since *G*. *aureus* are native to Taal, Naujan, and Buluan, and these lakes are large and still have a connection to the sea, these may explain why these populations were able to expand after a bottleneck event. These population expansions were also supported by the star-shaped haplotype networks detected for populations of *G*. *aureus* from Lake Taal and Lake Naujan (Fig 2), unimodal shaped mismatch plot for Lake Naujan, and non-significant SSD values and raggedness indices for both sudden and spatial expansion models for Lake Naujan and also for Lake Taal with the exception of the SSD value for sudden expansion model. High haplotype and low nucleotide diversities based on mtDNA *cyt b* were also observed in other fish species such as Chinese pomfret *Pampus chinensis* (*h* = 0.540 to 0.828, *π* = 0.081% to 0.295%) [86], the cyprinid *Schizopygopsis younghusbandi* (*h* = 0.922, *π* = 0.36%) [87], river goby *Glossogobius callidus* (*h* = 0.91, *π* = 0.4 to 0.5%) [88], and schizothoracine *Schizopygopsis younghusbandi* (*h* = 0.93, *π* = 0.33%) [38].

Both the haplotype and nucleotide diversities are considered low [83] for the native population in Laguna de Bay. The low genetic variation in this population is possibly due to bottleneck. Laguna de Bay has been reported to be ecologically stressed since the 1960s. Delmendo [89] reported that the lake was under high fishing pressure in the early 1960s. Other stressors that acted on the different populations of fish in Laguna de Bay include the use of destructive fishing gears, presence of pollutants, siltation, perennial high turbidity, and eutrophication [90]. These stressors accounted for the decline in open water fish harvest [90]. In addition, Pasig River, the only connection of Laguna de Bay to Manila Bay, was declared biologically dead in the 1980s [91] due to water quality degradation. This prevented the migration of organisms, including *G*. *aureus*, through the river. Laguna de Bay was then completely isolated; hence, the *G*. *aureus* population in the lake became landlocked as it had no opportunity to interact with other populations. This isolation prevented further gene flow between the population in Laguna de Bay and other populations.

Both the haplotype and nucleotide diversities are also considered low [83] for the native populations in Bato and Buhi lakes. Lake Bato and Lake Buhi populations could have experienced bottleneck due to the severe degradation of the lakes brought about by tilapia fish cage congestion [92, 93] and overfishing. *Glossogobius aureus* fry were possibly being caught indiscriminately along with another small goby species, the endemic *sinarapan Mistichthys luzonensis*. *Sinarapan* was recorded to have been overharvested from the 1960s to 1970s with the use of fine-meshed nets [94–96]. *Glossogobius aureus* population in Lake Buhi is represented only by one haplotype, Hap2, even though the samples used in this study came from different batches collected at different times. The presence of only one haplotype might be due to a combination of founder event and genetic bottleneck. Lake Buhi is only three centuries old [49]. It is possible that not many individuals were able to invade and proliferate in Lake Buhi and then got isolated, overfished and exposed to a stressful environment. In 1955, a dam was constructed at the lake’s outlet [94, 97], preventing migratory fish, including amphidromous *G*. *aureus*, from entering the lake, thus rendering the Lake Buhi’s *G*. *aureus* population landlocked. From 1975 to 1978, motorized push nets were being operated in Lake Buhi to increase the catch of *sinarapan* [94]. This fishing method involved the use of fine nets to maximize the fish catch, which resulted in overfishing not only of *sinarapan* but also of the fry of other fish species. The use of motorized push nets did not only result in overharvesting of the fishes in Lake Buhi but also disturbed the lake’s aquatic vegetation that served as spawning grounds and hiding places for goby species from predators [97]. In 2013, a pollutant-tolerant algal species *Aulacoseira granulata* was documented to be the dominant diatom species in Lake Buhi, indicating that the lake was under pollution stress [98]. Since 1980, recurring fish kills have been observed in Lake Buhi; a massive one occurred in September 1998 [93].

The population of *G*. *aureus* in Lake Sebu also had both low haplotype and low nucleotide diversities. This can be attributed to founder effect because of the high likelihood that *G*. *aureus* has been introduced into Lake Sebu. Taxonomic study on Lake Sebu fishes is scanty. The presence of *Glossogobius* species in Lake Sebu was only recorded in 2015 [81] and 2016 [82]. The low genetic diversity can also be due to bottleneck brought about by fish kills in 2017 [99], January 2021 [99] and January 2023 [100]. Specimens from Lake Sebu were collected in 2021 and just a few weeks after the 2023 fish kills.

Just like in Lake Buhi, the population of *G*. *aureus* in Lake Paoay is also represented only by one haplotype (Hap5) and consequently, the haplotype and nucleotide diversity levels are zero. The presence of only one haplotype in Lake Paoay population might be due to the combination of founder event and lack of gene flow [101]. The species was documented in the lake only in 1977 and was said to have been inadvertently introduced to Lake Paoay with other fish species [52]. Lake Paoay has a different drainage system; it is devoid of tributaries [52]. The presence of a single haplotype (Hap5) is a sign of fixation, which indicates that Lake Paoay population might have originated from just a small number of individuals. This population was also the most genetically distinct among the *G*. *aureus* populations, possibly due to isolation. In the study of Santos et al. [102] on the morphological variation of the Philippine endemic silver perch *Leiopotherapon plumbeus*, it was found that the population from Lake Paoay was also the most distinct and the authors attributed this to isolation.

This study revealed that genetic diversity is higher in native populations of *G*. *aureus* in Lake Taal, Lake Naujan, and Lake Buluan than in introduced populations in Lake Lanao and Lake Paoay. Introduced populations tend to exhibit low diversity when only few individuals, carrying small fraction of the original DNA composition of the source population [103], are translocated or when a large number of individuals are introduced, but only few get to survive and establish in the new environment [104]. Similar finding has been observed in economically important fish species such as catfish *Pangasius* sp. [27], striped snakehead *Channa striata* [28], European catfish *Silurus glanis* [30], and bighead carp *Hypophthalmichthys nobilis* [105]. Ha et al. [27] also found high genetic diversity in indigenous wild Bangladesh Pangas catfish *Pangasius pangasius* and low genetic diversity in introduced and hatchery-bred Thai and Vietnam striped catfish *Pangasius hypophthalmus*. Alam et al. [28] also found high genetic diversity in Bangladesh’s indigenous striped snakehead *Channa striata* populations and low diversity in introduced populations. Castagne et al. [30] also observed the same pattern in native (with high genetic diversity) and non-native (with low genetic diversity) populations of European catfish *Silurus glanis*. However, in certain cases, introduced populations exhibit higher genetic diversity than native populations. In this study, the native population in Laguna de Bay and Bato had lower genetic diversity than the introduced populations in Lanao and Sebu lakes. Xing et al. [29] also found that the introduced clearhead icefish *Protosalanx hyalocranius* population had higher genetic diversity than the native populations in China. Similarly, some of the introduced populations of European catfish *Silurus glanis* demonstrate higher genetic diversity than their native counterparts [30]. Multiple introductions from different distinct genetic sources were put forward to explain this observation [30, 106]. Similarly, it is possible that the introduction of *G*. *aureus* into Lanao and Sebu lakes happened more than once. This is supported by the existence of two haplogroups (Cluster A and Cluster C) of *G*. *aureus* in the Lanao and Sebu populations and the *G*.cf. *aureus* in Lake Lanao. Additionally, the suitable new habitat and the species’ ability to successfully establish itself in its new environment can contribute to high genetic diversity in the introduced population. This ability can be facilitated by the high plasticity of their life-history traits, high tolerance to adverse environmental conditions and high reproductive success. These were the explanations suggested by Agdamar and Tarkan [107] for the high genetic diversity in a non-native freshwater *Carassius gibelio* in Turkey.

The practice of introducing economically important fish to new regions dates back to as early as the 17^th^ century [107], with the aim of enhancing fishery resources [108]. However, only limited number of studies have evaluated the effect of such introduction on the genetic background of the introduced, source, and recipient populations and its consequences on the native conspecific fish populations [108]. Prior knowledge of the genetic diversity of both the source and recipient populations is necessary as the genetic consequence of the introduction varies depending on the background genetic information of the source and recipient populations. For example, the introduction of a population in a new environment may reduce the genetic diversity of the introduced population due founder effect and bottleneck. This is demonstrated in the case of the introduction of *Pangasius hypophthalmus* in Bangladesh [27]. When introduction is carried out to augment the existing population, it may increase the genetic diversity of the recipient population as seen in the wild Chinese sucker *Myxocyprinus asiaticus* in the Wanzhou reaches of upper Yangtze River [109]. Conversely, there are cases where introduced populations may displace native ones, as exemplified by the native bighead carp *Hypophthalmichthys nobilis* and silver carp *Hypophthalmichthys molitrix* populations in the Pearl River [108]. These native populations are currently at risk of being displaced by introduced populations from the Yangtze River [108]. In the case of the Philippine *G*. *aureus*, the introduced populations have low genetic diversity probably because of founder effect, isolation, and lack of deliberate effort to introduce *G*. *aureus* individuals from other genetically diverse sources. All the introduction events were accidental [52, 78] and as a consequence, only few individuals were most likely introduced.

### Genetic relatedness and divergence among the populations

The chi-square test for genetic differentiation, AMOVA, pairwise and overall *F*_ST_ values indicate pairwise genetic differentiation among all populations of *G*. *aureus* in this study except those from Laguna de Bay, Lake Bato, and Lake Buhi. Genetic divergence is expected among the different populations of *G*. *aureus* because they are landlocked freshwater gobies that thrive in lakes that are separated by physical barriers, such as terrestrial and aquatic barriers. Ward et al. [110] found a greater degree of genetic differentiation in freshwater fishes than in marine and anadromous fishes. This was attributed to the limited ability of freshwater fishes to migrate from one population to another because of the presence of more pronounced physical barriers among freshwater populations. The same pattern was also observed by Knight et al. [111] in Oxleyan pygmy perch *Nannoperca oxleyana* populations that thrive in areas that are separated by physical barriers such as sand dunes. This genetic differentiation may also explain why majority of the haplotypes in this study were not shared among the populations. A high genetic differentiation was also detected in different populations of other Philippine freshwater fishes, such as cyprinid *Barbodes tumba* [112] and the eleotrid *Giuris laglaizei* [113], which also thrives in different geographic regions.

The nonsignificant *F*_ST_ value observed among the *G*. *aureus* populations in Laguna de Bay, Lake Bato, and Lake Buhi suggests common ancestry in the past. This is supported by a shared haplotype (Hap2) among the three populations. In 1981, Laguna Lake Development Authority established lake-based hatchery and nursery facilities in Laguna de Bay [114]. Laguna and Rizal hatcheries primarily sold fingerlings to tilapia cage operators in Laguna de Bay, Lake Buhi, and Lake Bato in 1982 [115]. It is possible that the fry or fingerlings from these facilities were contaminated with fry or fingerlings of *Glossogobius* species as fish pens installed in Laguna de Bay served as breeding areas and refuges for other fishes, including *Glossogobius* [116]. The moderate genetic differentiation between Lake Buluan and Sebu populations could be attributed to the possibility that the population in Lake Sebu originated from Lake Buluan. It is also possible that both populations originated from a common source since both lakes are used for tilapia farming. Tilapia farmers were reported to procure tilapia seeds from hatcheries in Banga, Surallah, Norala and Marbel municipalities in South Cotabato [53]. It is possible that gene flow between the *G*. *aureus* populations in the two lakes was facilitated by stocking the lakes with tilapia seeds. Lake Sebu is represented by three haplotypes (Hap5, Hap25, and Hap26). Majority of the individuals (73%) belong to Hap5, which is shared with Lake Buluan population. Hap5 is also the most numerous (36.67%) among the five haplotypes in the Lake Buluan population.

Both the MJN (Fig 2) and ML tree (Fig 3) grouped the 29 haplotypes into four clusters. Three of these clusters had a star-like shape with Hap2, Hap7, and Hap18 as central common haplotypes. The star-like topology of the MJN is often viewed as evidence for recent population expansion [67]. This is supported by the mismatch analysis wherein the populations in Laguna de Bay and Naujan lakes have unimodal mismatch graphs. The most common haplotype (Hap2) was found in 107 individuals and was shared by all populations except those from Paoay and Sebu lakes. This indicates that it is among the ancestral haplotypes of the Philippine *G*. *aureus*.

It is worth noting that specimens from Lake Lanao are divided into two lineages, *G*. *aureus* and *G*. cf. *aureus*, that are genetically highly differentiated (*F*_ST_ = 0.983, *P*-value = 0) and are grouped into different clusters in the MJN (Fig 2) and ML tree (Fig 3). The presence of the two lineages along with the presence of two haplogroups (Cluster A and Cluster C) is an indication that *G*. *aureus* populations in Lake Lanao were introduced multiple times. *Glossogobius* cf. *aureus* was only detected in Lake Lanao among the Philippine lakes in a DNA barcoding study by Abdulmalik-Labe et al. [10]. It is possible that *G*. cf. *aureus* was brought to Lake Lanao in the 1950s when the then Philippine Fisheries Commission, now the Bureau of Fisheries and Aquatic Resources (BFAR), introduced milkfish larvae to Lake Lanao [78]. Based on the mtDNA *COI* sequences submitted to GenBank, *Glossogobius* cf. *aureus* were also detected in Taiwan (with nominal name *G*. *giuris* and GenBank accession no. KU944939), Pangasinan, Philippines (with nominal name *G*. *giuris* and GenBank accession no. KF714950), and San Jose, Antique, Philippines (with nominal name *G*. *aureus* and GenBank accession no. OQ387838). The percentage identity between our *G*. cf. *aureus* and these three sequences ranged from 99.84% to 100%. Taiwan and Philippines are among the leading countries in milkfish production [117]. The Philippines imports milkfish fry mainly from Indonesia and partly from Taiwan to augment local milkfish fry production. In addition to hatcheries, the milkfish industry in the Philippines also sources its fry supply from the wild. The Philippine milkfish fry gathering grounds are found on the western and southern coasts of the islands [118], which include Pangasinan and Antique. During the milkfish fry collection, other fish larvae and juveniles are also captured but discarded with the debris [117, 118]. However, milkfish fry are often confused with goby fry [117], and it is possible that the milkfish larvae introduced by the BFAR to Lanao Lake in the 1950s were contaminated with *G*. cf. *aureus*.

### Implications for conservation and management

The low levels of nucleotide diversity observed in all *G*. *aureus* populations highlight the need for immediate conservation intervention. The best conservation option for populations of *G*. *aureus* that are carnivorous [20], genetically diverged, and geographically separated is to protect them in their native habitats [119]. Conservation efforts must also address other threats like pollution, habitat fragmentation or loss, and introduction of invasive species. The presence of unique haplotypes in each population, in addition to the large genetic divergence between populations, indicates that each population must be managed separately. Management includes fishing regulation, habitat restoration, incorporation of active genetic management [120], and establishment of a refuge population [121]. Regulation of *G*. *aureus* fishing during the peak spawning season must be implemented. *Glossogobius aureus* is reported to have a higher spawning activity during the months of August to October [11].

Introduced or non-native populations of *G*. *aureus* in the Philippines exhibit low nucleotide diversity that ranges from 0.00% to 0.380%. These populations might benefit from augmenting or stocking individuals from closely related viable native or wild populations, preferably populations of higher genetic diversity [26]. This stocking approach may increase the genetic diversity of the recipient population [109] or at least increase the probability of the survival of the species [26]. However, caution must be observed as unknown threats may be introduced to the recipient population. If stocking is to be done, the following are recommended: (1) a taxonomic survey of existing ichthyofauna must be done before stocking to document any change in the species diversity resulting from the stocking activity; (2) careful stocking procedures must be practiced, ensuring that the only intended species, *G*. *aureus*, is being translocated; (3) systematic documentation of the stocking activity such as information on the origin, quantity, and genetic diversity information of the stock released to the recipient lake; and, (4) post-stocking monitoring and evaluation of the effect of the stocking activity on the genetic characteristic of the recipient population, as stocking activity can alter the genetic diversity of the recipient population [109]. Since the *F*_ST_ value between Laguna de Bay and Lake Bato populations is not significant, the two populations might benefit from reciprocal stocking. This will facilitate the transfer of unique haplotype from one lake to the other, thereby increasing the genetic diversity in each lake. Genetic diversity of Lake Buhi population can be increased if individuals from Laguna de Bay and Lake Bato are translocated into Lake Buhi, as the population of *G*. *aureus* in these three lakes are not genetically differentiated based on the results of this study.

## Conclusions

We used mtDNA *cyt b* in this study to investigate the pattern of genetic diversity, relatedness, and divergence among native and translocated populations of *G*. *aureus* in nine Philippine lakes. High haplotype and low nucleotide diversities were observed in the native populations of Taal, Naujan, and Buluan lakes. In contrast, low haplotype and low nucleotide diversities were observed for the native populations in Laguna de Bay, Bato, and Buhi lakes and introduced populations in Lanao, Paoay, and Sebu lakes. All the populations studied had low nucleotide diversity, indicating that these populations experienced bottleneck. Thus, this study calls for immediate conservation management intervention. Our results also showed genetic differentiation in majority of the populations. This indicates that each population must be managed separately, especially Taal, Lanao, Naujan, Buluan, Paoay and Sebu populations as these populations exhibit moderate to very high *F*_ST_ values. Since Lake Buhi and Lake Paoay populations are represented each by a single haplotype, individuals from other populations can be introduced into these lakes to increase their genetic diversity [122]. Populations from Laguna de Bay and Lake Bato can be sources of individuals that are to be introduced to Lake Buhi. Since populations from Taal, Naujan and Buluan lakes have the highest haplotype diversities, they can serve as sources of stock for introduction into other lakes. Genetic diversity and population structure of *G*. *aureus* in other water bodies and other countries must also be studied to widen the possible sources of broodstock if the fish is to be used for aquaculture.
